# Bileaflet mechanical aortic valves do not alter ascending aortic wall shear stress

**DOI:** 10.1007/s10554-018-1508-9

**Published:** 2019-02-11

**Authors:** Emile S. Farag, Emilio L. Schade, Pim van Ooij, S. Matthijs Boekholdt, R. Nils Planken, Roland van Kimmenade, Aart J. Nederveen, Bas A. J. M. de Mol, Jolanda Kluin

**Affiliations:** 1 Department of Cardiothoracic Surgery, Academic Medical Center, Amsterdam, The Netherlands; 2 Department of Radiology and nuclear medicine, Academic Medical Center, Amsterdam, The Netherlands; 3 Department of Cardiology, Academic Medical Center, Amsterdam, The Netherlands; 4 Department of Cardiology, Radboud Medical Center, Nijmegen, The Netherlands

**Keywords:** 4D flow MRI, Magnetic resonance imaging, Aortic valve prosthesis, Mechanical aortic valve, Wall shear stress

## Abstract

Progressive ascending aortic dilatation has been observed after mechanical aortic valve replacement (mAVR), possibly due to altered blood flow and wall shear stress (WSS) patterns induced by their bileaflet design. We examined the effect of mAVR on WSS in the ascending aorta using time-resolved 4D flow MRI. Fifteen patients with mechanical aortic valve prostheses, 10 patients with bicuspid aortic valve disease and 10 healthy individuals underwent thoracic 4D flow MRI. Peak systolic hemodynamic parameters (velocity and WSS) and vessel diameters were assessed in the ascending aorta. In addition, three-dimensional per-voxel analysis was used to compare velocity and WSS between patient groups and healthy controls. Peak aortic diameters were significantly higher in mAVR and BAV patients compared to healthy controls (p = 0.011). Mean aortic diameters were comparable between mAVR and BAV patients. No differences in 4D flow MRI-derived mean blood flow velocity and peak WSS were found between the three groups. Compared to healthy controls, mean WSS was significantly lower in mAVR patients (p = 0.031). Per-voxel analysis revealed no increased WSS in the ascending aortic wall and significantly lower velocity and WSS values in mAVR patients compared to healthy controls. In contrast, regions of significantly increased outer lumen velocities and WSS in BAV patients compared to healthy controls were found. This study shows that there is no increased ascending aortic WSS after mAVR. Our results suggest that, in contrast to BAV patients, there is no indication for intensified follow-up of the ascending aorta after mAVR.

## Introduction

The only definitive treatment for severe aortic valve disease is aortic valve replacement (AVR), which is performed by replacing the dysfunctional aortic valve by a either a mechanical heart valve or a bioprosthetic tissue valve [1]. Mechanical valves are made of pyrolytic carbon, have a bileaflet design and are designed to last a lifetime, but the effect of mechanical aortic valve replacement (mAVR) on progressive aortic dilatation is disputed [2, 3]. It is possible that their bileaflet design, in contrast to the trileaflet design of the native aortic valve or bioprosthetic aortic valves, induces changes in blood flow patterns and wall shear stress in the aortic root, resulting in progressive ascending aortic dilatation.

Time-resolved 3D phase contrast magnetic resonance imaging (MRI) with three-directional velocity encoding, also known as 4D-flow MRI, is a novel imaging modality capable of measuring blood flow in the three principal directions and as a function of time, which allows for the quantification of blood velocity in both the heart and the great vessels [4]. 4D-flow MRI can be used to calculate hemodynamic parameters in vivo, such as wall shear stress (WSS) [5, 6]. 4D flow MRI studies comprising bicuspid aortic valve (BAV) disease patients have shown that the abnormal valve morphology in BAV disease leads to increased WSS in the ascending aorta and that areas of increased WSS are subject to dysregulation of the extracellular matrix and degeneration of elastic fibers in the aortic wall, leading to aortic wall remodeling [7]. This finding implies an important role for the tricuspid architecture of the aortic valve in its hemodynamic performance. Therefore, hemodynamic performance of implanted bileaflet valve prostheses and their effect on remodeling are of interest for long-term prognosis because increased WSS may trigger aortic wall degradation and may result in aortic dilatation [8–10].

The aim of this study is to investigate blood flow and WSS patterns in the ascending aorta in patients with mechanical aortic valves and to determine whether mAVR leads to WSS deviations in the ascending aorta.

## Methods

### Study cohort

**Fig. 1:**
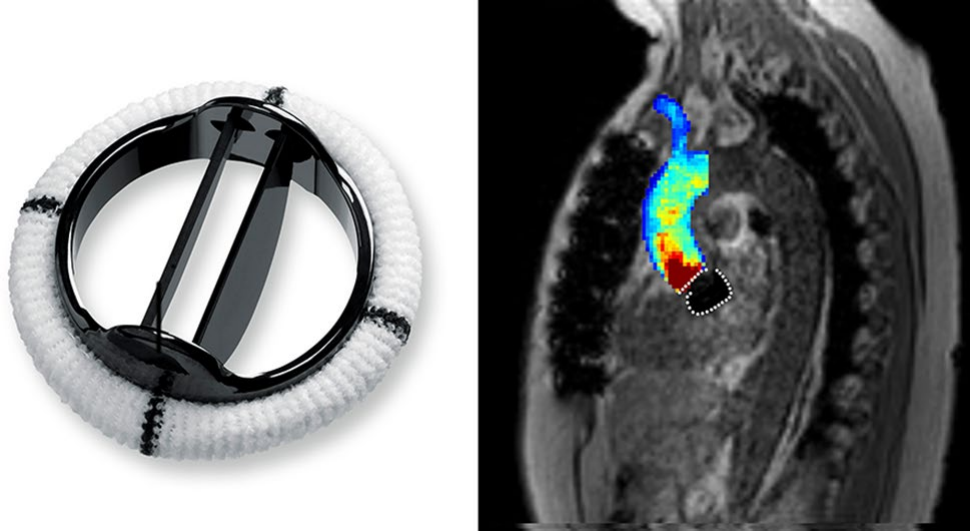
Examples of a mechanical aortic valve prosthesis (left, with permission of Abbott) and a velocity maximum intensity projection measured with 4D flow MRI (right). Marked white delineated area (right) represents susceptibility artifact caused by mechanical valve prosthesis

In this single center cross-sectional study, 15 patients who underwent mAVR between January 2010 and November 2015 were recruited (Fig. 1a). Patients having undergone concomitant other surgical procedures or having suffered from peri- or postoperative complications were excluded. Demographic data, surgical history data and cardiovascular risk factors were obtained from electronic medical records. Cardiac and aortic characteristics were retrieved from pre- and postoperative echocardiograms, which are defined as latest echo prior to surgery and latest echo prior to discharge. Echocardiograms were analyzed by an experienced cardiologist (SMB).

In addition, 10 age- and gender-matched patients with Sievers’ type 1 right-left cusp fusion BAV disease and normal aortic valve function (no aortic valve stenosis and/or regurgitation) and 10 healthy age- and gender-matched volunteers with no history of cardiovascular disease or surgery were enrolled. Patients with contra-indications for MRI and/or older than 65 years of age were excluded for study participation. The institutional review board approved the study and all patients provided signed informed consent.

### Magnetic resonance imaging

All participants underwent cardiac and respiratory-gated sagittal 4D flow MRI of the thoracic aorta on a 3.0 T MRI scanner (Ingenia 3.0T MR System, Philips Healthcare, Eindhoven, The Netherlands). Standard transmit and receive cardiac coils were used for 4D flow measurements. 4D flow MRI sequence parameters were the same for all subjects: spatiotemporal resolution: 2.5 × 2.5 × 2.5 mm^3^, ± 42 ms (24 timeframes per heart cycle); TE/TR/FA = 2.1 ms/3.4 ms/8°; VENC: 150–250 cm/s; k-t PCA acceleration factor: 8; scan time approximately 7 min [11]. A field of view covering the entire thoracic aorta was defined and data was collected throughout the heart cycle in ± 300 heartbeats, including both systolic and diastolic timeframes.

The ascending aorta (defined as the aorta between the aortic valve and the origin of the brachiocephalic trunk) was segmented (Mimics, Materialise, Leuven, Belgium) using 3D phase contrast MR angiography images, created by multiplication of the magnitude with the absolute velocity images followed by averaging over all timeframes. MRI data were corrected for eddy currents, Maxwell terms and velocity aliasing using in-house software programmed in MatLab (MathWorks, Natick, MA, USA). The peak systolic time frame was defined as the time frame with the highest velocity averaged over the segmentation. Mean and maximum blood flow velocities in the ascending aorta were calculated at the peak systolic time frame, as measured using simultaneous and triggered electrocardiography (Fig. 1b) [12]. Aortic mean and peak wall shear stress (WSS) was calculated with a previously published algorithm [13].

### Cohort-averaged velocity and WSS maps

Comparison of local differences in velocity and WSS was conducted by cohort-averaging individual maps using a shared geometry method developed using in-house software in Matlab, as previously described [14, 15]. A shared geometry representing all aortic shapes for each group was generated. Next, individual aortic segmentations were registered to the shared geometry followed by interpolation of the individual velocity and WSS values to the shared geometry. After averaging groups separately, cohort-averaged 3D maps for WSS and peak systolic velocity were obtained and displayed. Peak systolic velocity was extracted from a maximum intensity projection (MIP) of the absolute velocity at peak systole [14, 15].

### Statistical analysis

Continuous variables with a normal distribution are reported as the mean ± standard deviation (SD) and continuous variables with a non-normal distribution are reported as median (interquartile range), and were compared using the Mann–Whitney U-test. Results were tested for Gaussian distribution using the Kolmogorov–Smirnov test. Categorical variables are reported as number and percentage. The Fischer exact test was used to compare nominal variables. Two-group comparisons were performed with paired or unpaired t-tests. For multiple group comparisons, the analysis of variance (ANOVA) method with Bonferroni post hoc analysis was performed. All statistical tests were two-sided and a p-value of 0.05 or lower was considered statistically significant. Statistical analyses were performed using the Statistical Package for Social Sciences (SPSS) software for Windows, version 22.0 (SPSS, Chicago, IL, USA).

Statistical local-differences analysis between groups was performed with per-voxel analyses using P-value maps as previously described [16]. In brief, a Wilcoxon rank sum test in every voxel (for velocity) and every point all the wall (for WSS) between the two groups was conducted. Subsequently, regions in which velocity/WSS is higher are delineated in red, regions in which velocity/WSS are lower are delineated in blue and regions with no significant differences are delineated in gray. Significant differences in velocity between cohorts were expressed as the vessel volume with significantly different velocity as a percentage of vessel volume of the complete ascending aorta. Significant differences in WSS between cohorts were expressed as the vessel surface with abnormally elevated WSS as a percentage of the vessel surface of the complete ascending aorta.

## Results

### Study cohort demographics

In total, 15 patients with a mechanical aortic valve prosthesis were included. Native aortic valves were tricuspid in all patients. Baseline characteristics are displayed in Table 1. Perioperative data and early surgical outcomes of mAVR patients are presented in Table 2. Furthermore, 10 patients with right-left fusion Sievers’ type 1 BAV disease and no signs of aortic dilatation and/or aortic valve dysfunction (stenosis and/or regurgitation) and 10 healthy individuals were prospectively enrolled.

**Table 1:** Baseline-characteristics

	mAVR	BAV	Control	p Value
N	15	10	10	
Age	54 ± 8	51 ± 8	52 ± 9	0.605
Female	2 (13)	3 (30)	4 (40)	0.306
Body mass index, kg/m^2^	27.7 ± 4.9	25.3 ± 4.0	26.6 ± 3.3	0.395
Body surface area, m^2^	2.4 ± 0.3	2.4 ± 0.2	2.4 ± 0.2	0.885
Hypertension	6 (40)	6 (60)	0 (0)	0.089
Diabetes mellitus	2 (13)	1 (10)	0 (0)	0.531
Surgical indication				
Aortic valve stenosis + regurgitation (%)	7 (47)	–	–	
Aortic valve insufficiency (%)	3 (20)	–	–	
Endocarditis	5 (33)	–	–	

Data are presented as mean ± standard deviation or number (percentage)

**Table 2:** Perioperative data and early outcomes of mAVR study participants

	mAVR (n = 15)
Time after surgery, years	3.2 ± 2.9
Valve prosthesis	
St Jude Medical HP	7 (47)
ON-X	4 (27)
Sorin bicarbon slimline	4 (27)
Prosthesis size (mm)	
21	2 (13)
23	6 (40)
25	6 (40)
27	1 (7)
Cardiopulmonary bypass time, min	97 ± 18
Cross-clamp time, min	70 ± 14
Postoperative peak velocity, m/s^a^	2.17 ± 0.52
Postoperative peak pressure gradient, mmHg^a^	20 ± 9
Postoperative mean pressure gradient, mmHg^a^	12 ± 6

Data are presented as mean ± standard deviation or number (percentage)

^a^Acquired by echocardiography

No significant differences were noted in age at the time of MRI between the three groups (mAVR, 54 ± 8 years; BAV, 51 ± 8 years; control, 52 ± 9 years; p = 0.605). Also, gender distribution did not differ significantly between groups (mAVR, 2 of 15 women; BAV, 3 of 10 women; control; 4 of 10 women; p = 0.306).

### Ascending aortic blood flow velocity, WSS and diameters

No statistically significant differences were found between mAVR and BAV patients in 4D flow MRI derived peak and mean ascending aortic diameters (mAVR vs. BAV: 43.1 ± 7.2 mm vs. 44.4 ± 8.7 mm (p = 1.000) and 35.6 ± 5.4 mm vs. 36.6 ± 6.5 mm (p = 1.000), respectively). However, compared to healthy controls maximum aortic diameters were significantly larger in both mAVR and BAV patients (p = 0.011). Mean aortic diameters were significantly larger in BAV patients compared to healthy controls (36.6 ± 6.5 mm vs. 30.3 ± 3.6 mm, p = 0.033).

Due to susceptibility artefacts caused by the titanium mechanical valve at the level of the aortic valve, 4D flow MRI assessment of peak aortic valve blood flow velocity was not possible in mAVR patients (Fig. 1b). However, peak blood flow velocity measured using transthoracic echocardiography (TTE) was 2.37 ± 0.48 m/s for mAVR patients and 1.87 ± 0.43 for BAV patients (p = 0.015). 4D-flow MRI derived mean blood flow velocity in the entire ascending aorta was 0.45 ± 0.13 m/s in mAVR patients, compared to 0.52 ± 0.09 m/s in BAV patients and 0.55 ± 0.11 m/s in controls (p = 0.078).

**Fig. 2:**
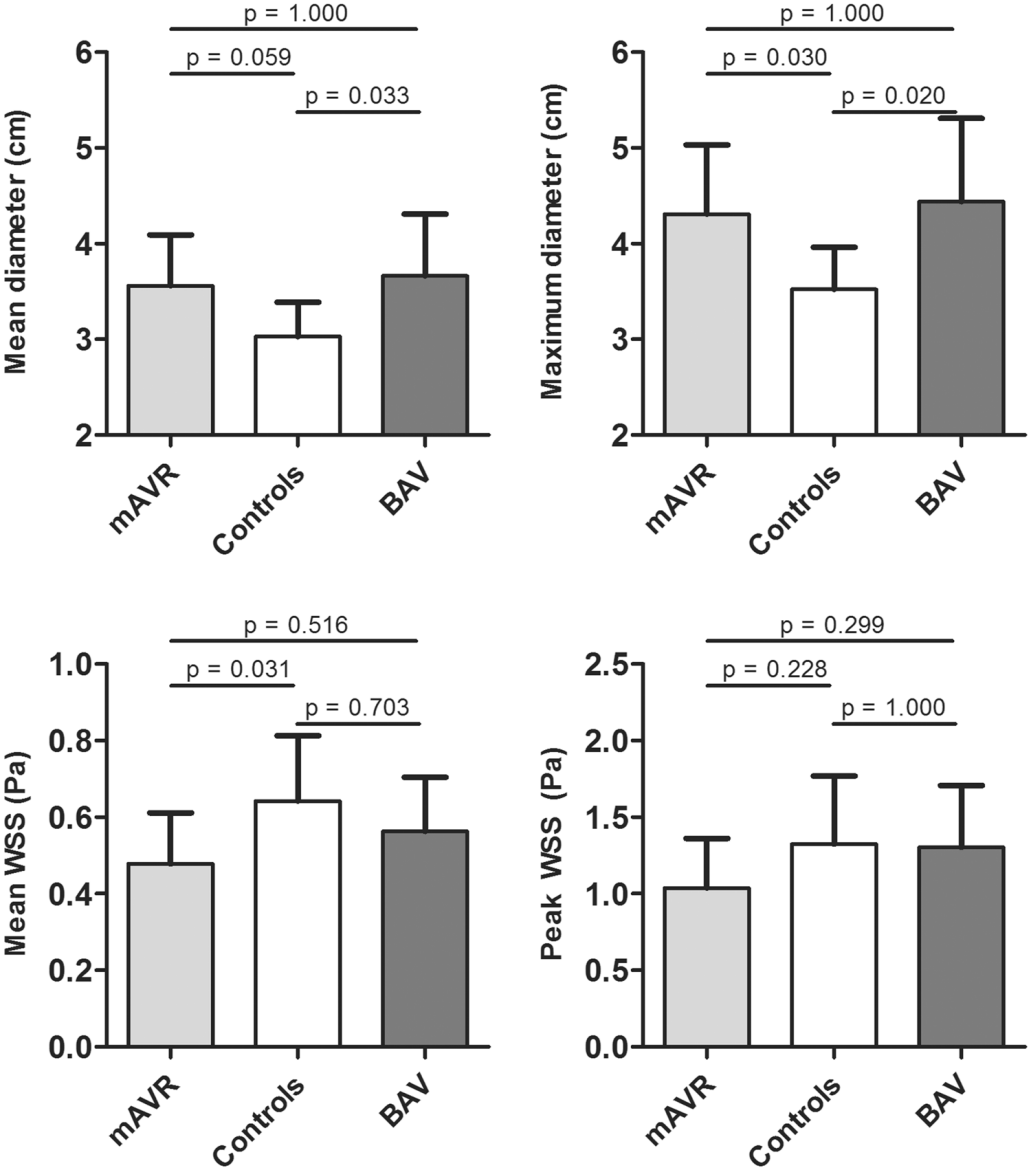
Quantitative analysis of 4D flow MRI derived mean and peak WSS and ascending aortic diameters. All p-values generated by the post-hoc Bonferroni test

Peak WSS in mAVR patients was 1.04 ± 0.32 Pa, comparable to 1.30 ± 0.40 Pa in BAV patients and 1.33 ± 0.45 Pa in controls (p = 0.123, Fig. 2). Mean WSS throughout the ascending aorta in mAVR patients was 0.48 ± 0.13 Pa, comparable to BAV patients (0.56 ± 0.14 Pa, p = 0.516) and lower than in healthy controls (0.64 ± 0.17 Pa, p = 0.031, Fig. 2). No differences were found between various valve prosthesis types and sizes. No significant correlation was found between maximum aortic diameter and peak WSS among mAVR patients (p = 0.573).

### Per-voxel analysis

**Fig. 3:**
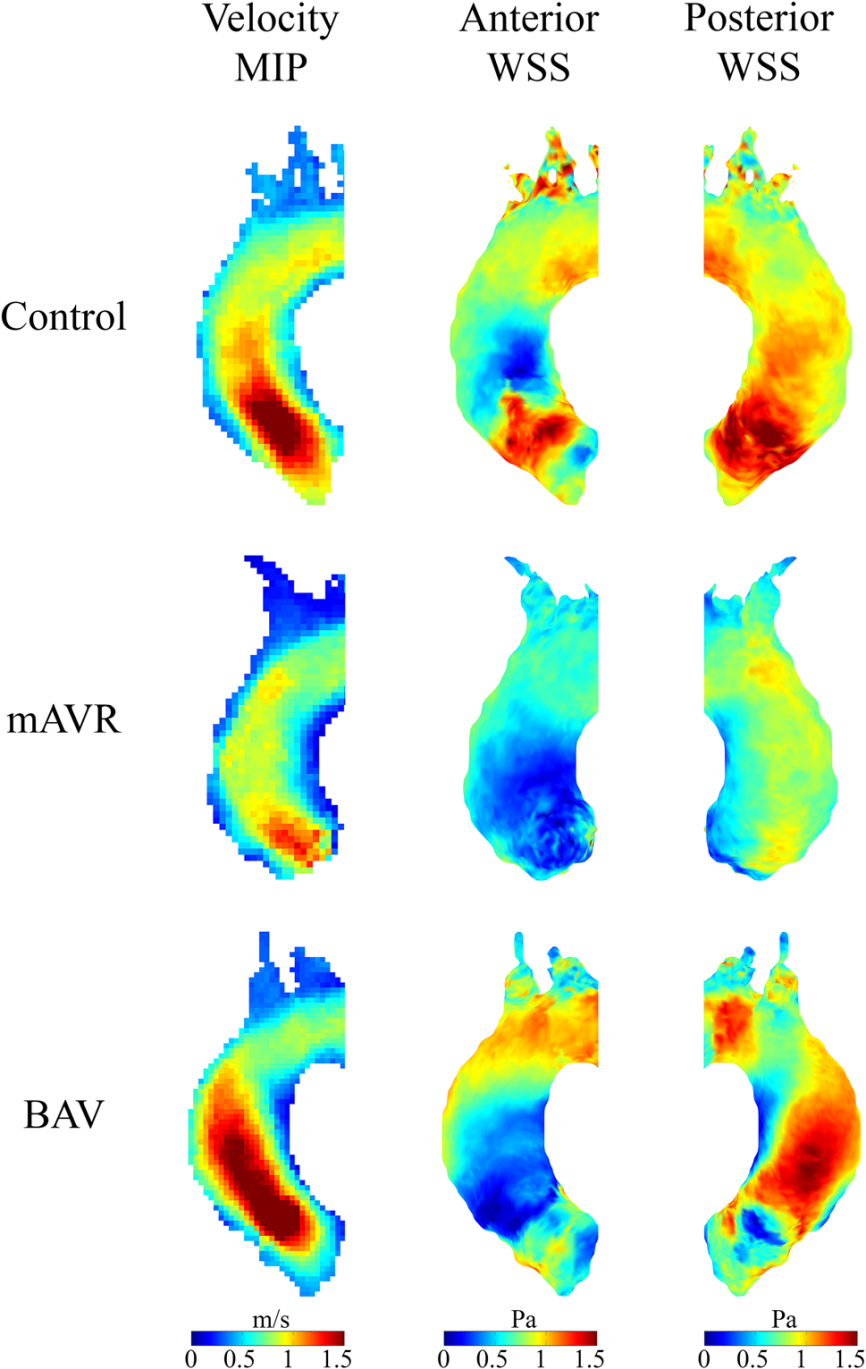
Cohort-averaged 3D maps for velocity maximum intensity projection (MIP) and wall shear stress (WSS) for healthy controls, mAVR and BAV patients displayed in grouped shared geometries. 4D flow MRI measurements were not possible at the level of the aortic valve in mAVR patients due to susceptibility artefacts caused by the titanium mechanical valve prosthesis

Differences in peak systolic velocity patterns and anterior and posterior WSS distribution between groups are displayed in cohort-averaged 3D maps in Fig. 3. The velocity MIP jet stream for mechanical valves at the level of the aortic root could not be visualized due to susceptibility artefacts caused by the titanium valve prosthesis (Fig. 1b). Compared to healthy controls, velocity jets for BAV patients were both wider at the level of the aortic valve and longer, directed towards the outer curvature of the ascending aorta.

**Fig. 4:**
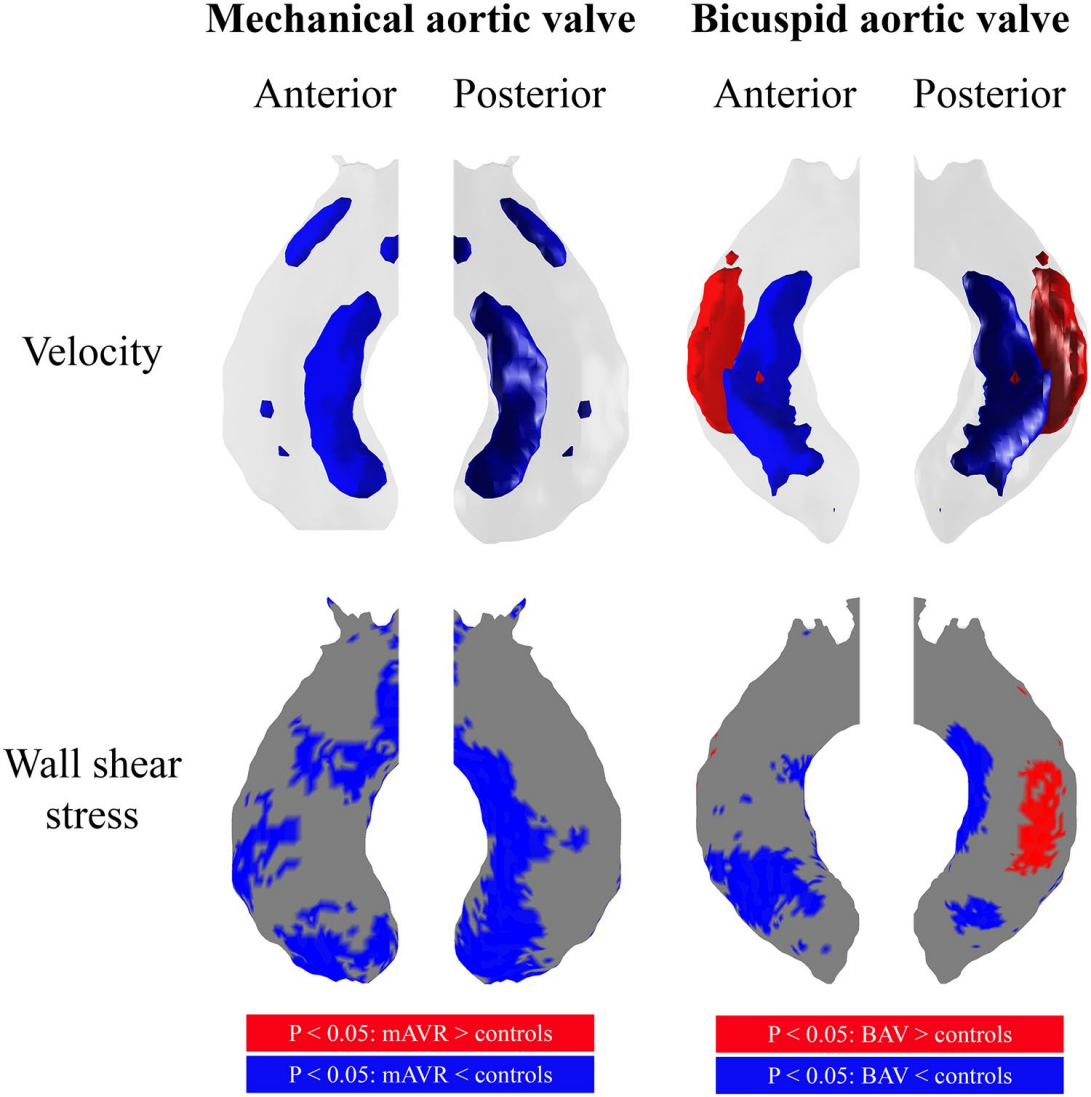
Ascending aortic p-value maps displayed in shared geometries from the anterior and posterior, displaying the significant differences for velocity and WSS between healthy controls and mAVR patients (left) and healthy controls and BAV patients (right). Red areas indicate significantly higher values for patient groups and blue areas indicate significantly lower values for patient groups

Per-voxel analysis using p-value maps (Fig. 4) comparing study groups with healthy controls revealed no increased velocity and/or WSS in mAVR patients throughout the ascending aorta. However, significantly higher velocities were found at the outer lumen of the greater curvature of the ascending aorta in BAV patients (significantly higher velocity in 8% of the vessel lumen of the shared geometry). This resulted in a region of increased WSS along the outer curvature of the ascending aorta compared to healthy controls (significantly higher WSS in 3% the vessel wall of the shared geometry).

Furthermore, inner curvature lumen velocities were significantly lower in mAVR patients compared to healthy controls (in 8% of the total vessel lumen) and WSS was significantly lower in 31% of the ascending aortic wall in mAVR patients. Similar to mAVR patients, lower central lumen velocity (9%) was present in the ascending aorta of BAV patients, resulting in 15% of the aortic wall subject to significantly lower WSS.

## Discussion

Altered aortic hemodynamics in BAV patients and the occurrence of aortic dilatation after mAVR have raised the question as to whether mechanical aortic valves exhibit BAV-like hemodynamic characteristics due to their bileaflet design. We have employed 4D flow MRI to assess blood flow velocity and WSS patterns in patients with a mechanical aortic prosthesis and compared them to patients with BAV and healthy individuals. We found that there is no increased WSS in the ascending aorta after implantation of a bileaflet mechanical aortic valve.

The effect of mAVR on blood flow has previously been studied using various imaging modalities. In-vitro and in-vivo studies have focused mainly on the effect of mAVR on coronary artery perfusion and have found that implantation and the specific orientation of mAVRs (relative to the native architecture of the aortic root and sinuses) influences aortic blood flow and coronary perfusion. Furthermore, one 4D flow MRI study, v on Knobelsdorff-Brenkenhoff et al. [10] found that in a cohort of 9 mAVR patients, mAVR resulted in increased blood flow vorticity in the ascending aorta. As a result, they found significantly lower peak WSS values in the distal ascending aorta compared to healthy individuals. These findings are in agreement with our results, showing comparable peak WSS values throughout the entire ascending aorta, but significantly lower mean WSS values. Furthermore, our per-voxel analysis comparing mAVR patients with healthy controls showed no regions of increased velocity and/or WSS, but large areas of significantly lower WSS in the ascending aorta. This finding suggests that, unlike BAV patients, patients after mAVR are not at risk of extracellular matrix dysregulation and elastic fiber degeneration due to their bileaflet design, ultimately resulting in progressive aortopathy [7].

We compared blood flow patterns after mAVR with both tricuspid and bicuspid aortic valves because the relationship between BAV disease and ascending aortopathy has been well established and has led to BAV-specific follow-up and treatment strategies in adult cardiac care [17]. In a recent 4D flow MRI study, Shan et al. found that abnormal blood flow patterns and WSS are present in BAV despite normal aortic valve function in patients with comparable aortic dimensions, resulting in increased peak systolic WSS compared to tricuspid aortic valves in the proximal ascending and mid-ascending aorta. This difference was even more pronounced in the presence of severe aortic valve stenosis and/or regurgitation [18]. Recent studies have also studied the relationship between aortic diameters ad WSS values, showing a significant correlation between aortic diameters and WSS measurements [19, 20]. Furthermore, a study comprising 25 patients has shown that patterns of aortic dilatation in BAV patients correspond with typical flow displacement patterns in various BAV subtypes, which may be the result of WSS-induced dysregulation of the extracellular matrix and degeneration of elastic fibers, as shown in previous studies [7, 21, 22].

Although we found no significant differences in mean and peak WSS throughout the entire ascending aorta due to the difference in mean and maximum aortic diameters between controls and BAV patients, our per-voxel analysis using p-value maps was in agreement with these previous studies and showed that regions of increased WSS are present in the wall of the outer curvature of the ascending aorta, caused by increased blood flow velocity in the outer lumen of the ascending aorta.

The difference in velocity MIP patterns between mAVR and BAV patients, as shown in the shared geometry velocity MIPs in Fig. 3 and the per-voxel analysis in Fig. 4, reflects the difference in the distortion of WSS patterns between both study groups. Presence of a Sievers’ type 1 right-left fusion BAV leads to increased blood flow velocity in the ascending aorta, resulting in increased WSS along the outer curvature of the ascending aorta. Furthermore, degeneration of a native BAV may lead to aortic valve stenosis and/or regurgitation, resulting in more distortion of flow and subsequently further alteration of WSS [19]. Our study shows that adequate implantation of a mechanical aortic valve does not induce such accelerated aortic blood flow resulting in increased WSS. In contrast to the asymmetrical opening of a Sievers’ type 1 BAV during systole, mechanical aortic valves consist of two symmetrical leaflets and thus resemble the orifice orientation of a “true” bicuspid aortic valve (Sievers type 0), leading to dissimilar flow patterns. Remarkably, previous findings demonstrate that, after valve sparing aortic root replacement, Sievers’ type 0 lateral BAVs exhibit ascending aortic WSS patterns similar to tricuspid aortic valve subjects, whereas Sievers’ type 1 right-left fusion exhibited higher WSS ratios along the outer ascending aortic curvature [23].

### Decreased velocity and WSS

It must be noted that per-voxel analysis also resulted in areas of decreased WSS in mAVR and BAV patient groups, which can be explained by two factors. First, WSS may have been affected by the difference in aortic dimensions between mAVR and BAV patient groups and healthy controls. Aortic diameters in healthy controls were significantly lower, which may have resulted in relatively higher WSS values throughout the ascending aorta [19, 20, 24]. Second, flow obstruction and increased vorticity, as demonstrated by van Knobelsdoff-Brenkenhoff et al. in mAVR patients, may result in viscous energy loss at the level of the aortic valve altering WSS [25].

### Limitations

Clinical implications of this study are limited by several factors. First, the present study is based on a relatively small cohort and therefore could be subject to selection bias for mAVR patients who underwent successful and uncomplicated valve replacement. Second, all mAVR patients underwent mAVR of a native tricuspid aortic valve, limiting the clinical representativeness of these findings for mAvR patients with a native BAV. Third, because of susceptibility artifacts caused by mechanical prostheses, comparison of the velocity jet and viscous energy loss at the level of the aortic valve was not possible. Finally, 4D flow data processing requires the semi-manually reconstruction of the acquired data, and these processing steps are prone to subjectivity that could affect the accuracy and interpolation of the 4D flow data. However, reproducibility and inter-observer variability of this processing method were previously investigated and show reproducibility and limited inter-observer variability [26]. Also, the spatial resolution used in this study were similar to other 4D flow MRI studies [7, 27].

## Conclusion

The present study shows that there is no BAV-like increased WSS in the ascending aorta after implantation of a bileaflet mechanical aortic valve prosthesis. Our results suggest the bileaflet design of a mechanical aortic valve prosthesis does not indicate intensified follow-up of the ascending aorta.
